# A majority of rural emergency departments in the province of Quebec use point-of-care ultrasound: a cross-sectional survey

**DOI:** 10.1186/s12873-015-0063-0

**Published:** 2015-12-11

**Authors:** Pierre Léger, Richard Fleet, Julie Maltais- Giguère, Jeff Plant, Éric Piette, France Légaré, Julien Poitras

**Affiliations:** Department of Family and Emergency Medicine, Université Laval, Quebec City, QC Canada; Research Centre, Emergency Medicine Laval University – CHAU Hôtel-Dieu de Lévis Hospital, 143 Wolfe Street, Lévis, QC G6V 3Z1 Canada; Faculty of Medicine, University of British Columbia, Vancouver, BC Canada; Department of Emergency Medicine, Penticton Regional Hospital, Penticton, BC Canada; Department of Family and Emergency Medicine, Université de Montréal; Department of Emergency Medicine, Hôpital du Sacré-Coeur de Montréal, Montréal, QC Canada; Department of Family Medicine and Emergency Medicine, Knowledge Transfer and Health Technology Assessment of the CHUQ Research Centre (CRCHUQ, Unité de Recherche Évaluative, Université Laval; Implementation of Shared Decision Making in Primary Care, Quebec City, QC Canada

## Abstract

**Background:**

Point-of-care ultrasound (POCUS) can be used to provide rapid answers to specific and potentially life-threatening clinical questions, and to improve the safety of procedures. The rate of POCUS access and use in Canada is unclear. The objective of this study was to examine access to POCUS and potential barriers/facilitators to its use among rural physicians in Quebec.

**Methods:**

This descriptive cross-sectional study used an online survey. The 30-item questionnaire is an adapted and translated version of a questionnaire used in a prior survey conducted in rural Ontario, Canada. The questionnaire was pre-tested for clarity and relevance. The survey was sent to non-locum physicians working either full- or part-time in rural emergency departments (EDs) (*n* = 206). All EDs were located in rural and small towns and provided 24/7 medical coverage with acute care hospitalization beds.

**Results:**

In total, 108 surveys were completed (participation rate = 52.4 %). Of the individuals who completed surveys, ninety-three percent were family physicians, and seven percent had Canadian College of Family Physicians – Emergency Medicine (CCFP-EM) certification. The median number of years of practice was seven. A bedside ultrasound device was available in 95 % of rural EDs; 75.9 % of physicians reported using POCUS on a regular basis. The most common indications for POCUS use were to rule out abdominal aortic aneurysm (70.4 %) and to evaluate presence of free fluid in trauma and intrauterine pregnancy (60 %). The most common reason (73 %) for not using POCUS was limited access to POCUS training programs. Over 40 % of POCUS users received training in POCUS during medical school or residency. Sixty-four percent received training from the Canadian Emergency Ultrasound Society, 13 % received training from the Canadian Association of Emergency Physicians, and 23 % were trained in another course. Finally, 95 % of respondents reported that POCUS skills are essential for rural ED practice.

**Conclusions:**

POCUS use in rural EDs in the province of Quebec appears to be relatively widespread. Access to training programs is a barrier to greater use.

## Background

Emergency department (ED) point-of-care ultrasound (POCUS) can be performed rapidly at patients’ bedside. POCUS results can respond to specific and potentially-life threatening clinical questions. Furthermore, compliance with POCUS procedures improves patient safety [1, 2]. POCUS is a safe and relatively low-cost technology [1]. For the focused scans often used in emergency practice, the learning curve for POCUS is not considered steep [1, 2]. Its use improves diagnostic accuracy and timely management of selected urgent conditions [3]. Many international medical associations have already endorsed POCUS as a standard of care [4, 5] and recommend training in POCUS as part of residency training and continuing medical education [6–9].

In 2006 and 2012, the Canadian Association of Emergency Physicians (CAEP) highlighted the importance of providing 24/7 access to POCUS in EDs nationwide [10, 11]. Despite the CAEP position on POCUS and its potential benefits, the statistics on actual use of POCUS in Canada are not clear [10, 11]. The literature in this area suggests that POCUS is increasingly taught and used in academic EDs in Canada [12, 13]. However, with the exception of one recent study conducted in Ontario [14], the use of POCUS in rural EDs has not been studied.

Rural EDs have limited local access to advanced imaging techniques such as computed tomography (CT) and formal ultrasound, and inter-facility transfers are often required to pursue diagnostic evaluations [15–18]. However, inter-facility transfer processes in rural and isolated areas can be costly, time-consuming and risky for patients and paramedics [19, 20]. POCUS has the potential to improve triage of patients requiring emergency inter-facility transfers, and to consequently improve access to timely care [1].

According to Flynn et al*.* [14], POCUS is not used to full potential in rural settings in Ontario. Flynn et al*.* [14] reported that only 60 % of their sample of 200 rural physicians had access to a bedside ultrasound device. Furthermore, only 44.4 % of physicians reported having the necessary knowledge or skills to perform POCUS. However, over 70 % of the sample stated that POCUS competence was an essential skill for the practice of rural emergency medicine [14]. The Flynn et al*.* study is recent and provides essential information; however, the low response rate (28.4 %) limits interpretation of the results, and further study of POCUS use in rural settings is warranted. The present study was designed to examine access to POCUS and potential barriers/facilitators to POCUS use among rural emergency physicians in Quebec.

## Methods

This descriptive cross-sectional study used an online survey (SurveyMonkey Inc, Palo Alto, California, USA). The study was added *a posteriori* to the ongoing Quebec Rural Emergency Department Project [15]. The research ethics committee of the “Centre de Santé et des Services Sociaux (CSSS)” Alphonse-Desjardins hospital approved the amendment to the project. The study’s objectives and the procedure were explained in the questionnaire. All respondents agreed to participate freely to the study by checking “yes” on the questionnaire.

### Selection of rural EDs and participants

Complete details on the definition of rural EDs and selection methods for the EDs included in the present study are provided elsewhere [15]. In brief, the Canadian Healthcare Facilities Guide was used to identify rural EDs providing 24/7 physician coverage and located in hospitals with acute-care hospitalization beds. We used Statistics Canada’s definition of rural and small towns to select communities to participate [21]. In total, 26 EDs met our inclusion criteria; of the 26, 19 agreed to participate in the study. To be eligible to participate, emergency physicians had to work for at least one of the 19 participating rural EDs.

### Data collection

The survey used in the Flynn et al*.* study [14] was translated into French, modified and adapted as per the literature on the subject. In compliance with recent survey design guidelines [22], the questionnaire was pre-tested for question clarity and item relevance on a convenience sample of ten emergency medicine residents from the Laval University Royal College of Physicians and Surgeons of Canada (FRCP). Individuals in the convenience sample had advanced training in POCUS, and their suggestions were used to modify and clarify the survey questionnaire. The final version of the survey included 30 questions. Eleven of the 30 questions pertained to socio-demographic information, including age, gender, level of medical training, year of graduation, number of years in practice, number of years practicing in the present community and distance to the nearest referral center. Two questions inquired about the availability of advanced imaging services. Seventeen questions addressed access and use of POCUS, including frequency of POCUS use, reason for POCUS use, and training in POCUS in medical school. The survey consisted of yes or no questions, multiple-choice questions and open-ended short answer questions. Questionnaire completion time was estimated to be less than ten minutes.

Data was collected between April 2013 and August 2013. The online survey (Survey Monkey software) was sent via e-mail to all part-time and full-time emergency staff practicing (*n* = 206) in the 19 selected rural EDs. To enhance participation, four automated reminder e-mails were sent at two-week intervals. Emergency department chairs were subsequently contacted by phone or e-mail and asked to remind colleagues of the study and to encourage participation.

### Statistical analysis

Data was imported into Microsoft Excel from the Survey Monkey software. We used simple descriptive analysis and calculated percentage and median. Data were analyzed with Excel (Microsoft Office) 2010.

## Results

In total, 108 of 206 eligible physicians participated in this study (participation rate = 52.4 %) Respondents’ descriptive characteristics are reported in Table 1. Briefly, 54.6 % of respondents were male, median age was 37 years and median year of graduation was 2006.

**Table 1:** General characteristics of physicians

Characteristics	Median or (%) *n* = 108
Gender (*n* [%])	
Male	59 (54.6)
Female	49 (45.4)
Age	
Median (range)	37 (25–62)
Medical training *n* (%)	
CCFP	100 (92.6)
CCFP (EM)	7 (6.5)
FRCP (EM)	0 (0)
Other	1 (0.9)
Years in practice	
Median (range)	7 (<1-34)
Years practicing in present community	
Median (range)	5.5 (<1-34)
Year of graduation	
Median (range)	2006 (1979–2013)
Distance to nearest referral center (km) (*n* [%])	
< 50	10 (9.3)
50-90	20 (18.5)
100-199	36 (33.3)
> 200	42 (38.9)

*No.* number, *CCFP* certification in Family Medicine from the College of Family Physicians of Canada, *CCFP (EM)* certificate in special competence in emergency medicine from the College of Family Physicians of Canada, *FRC(EM)* Royal College of Physicians and Surgeons of Canada (Emergency Medicine Board Certification)

Table 2 presents the general characteristics of the EDs in the study. In summary, 95.4 % of respondents reported access to a POCUS device. Most EDs had over 10,000 visits per year. The majority of participating EDs (78 %) had local 24/7 access to CT scanners.

**Table 2:** Rural ED characteristic

Variables	*n* (%)
ED shifts per year	
< 40	5 (4.6)
41–80	32 (29.6)
81–120	38 (35.2)
121–160	24 (22.2)
> 160	9 (8.3)
ED volume, patients per year	
< 5,000	13 (12.0)
5,000–9,999	13 (12.0)
10,000–14,000	25 (23.1)
15,000–19,000	22 (20.4)
> 20,000	35 (32.4)
CT scanner in hospital (24/7 access)	
Yes	85 (78.7)
No	23 (21.3)
Ultrasonography in hospital (performed by radiologist)	
Yes, every day	17 (15.7)
Yes, sometimes	80 (74.1)
No	11 (10.2)
POCUS device available in ED	
Yes	103 (95.4)
No	5 (4.6)

*No.* number, *ED* emergency department, *CT* computed tomography, *POCUS* point of care ultrasound

Seventy-six percent of physicians reported POCUS use (Table 3). The two most common reasons for use were to rule out an abdominal aortic aneurysm (70.4 %) and to verify the presence of free fluid in the abdomen (70.4 %). Seventy-three percent of physicians who reported not using POCUS cited lack of training as the primary reason. Over 40 % of POCUS users reported having been trained in POCUS during their academic medical training. Sixty-four percent of trained users received training through the POCUS course offered by the Canadian Emergency Ultrasound Society (CEUS). Finally, half of non-trained physicians cited long waiting lists as the primary reason for not obtaining training in POCUS.

**Table 3:** Physician POCUS use

Variables	n (%)
Use POCUS	
Yes	82 (75.9)
No	26 (24.1)
Frequency of POCUS use	
< 1 per week	42 (38.9)
Once per ED shift	24 (22.2)
> 1 per ED shift	15 (13.9)
Never	27 (25.0)
Reasons for POCUS use	
Ruling out AAA	76 (70.4)
Ruling in intrauterine pregnancy	69 (63.9)
Ruling out peritoneal free fluid	76 (70.4)
Ruling out pericardial effusion	66 (61.1)
Central line placement	17 (15.7)
Do not use POCUS	27 (25.0)
Other	15 (13.9)
Do not perform POCUS because of … (*n* = 37)	
Lack of training	27 (73.0)
Difficulty maintaining skills	12 (32.4)
Lack of need	4 (10.8)
Cost	0 (0.0)
Other	5 (13.5)
Training in POCUS during medical school	
Yes	44 (40.7)
No	64 (59.3)
Where were you trained in POCUS? (*n* = 86)	
CAEP	11 (12.8)
CEUS	55 (64.0)
Other	20 (23.2)
Difficulty obtaining POCUS training because… (*n* = 52)	
Long waiting list	26 (50.0)
Distance to training center	22 (42.3)
Other	14 (26.9)
POCUS is a skill that an EP should have	
Strongly agree	72 (66.7)
Agree	31 (28.7)
Neither agree nor disagree	5 (4.6)
Disagree	0 (0.0)
Strongly disagree	0 (0.0)
POCUS is a skill that a rural EP should have	
Strongly agree	67 (62.0)
Agree	36 (33.3)
Neither agree nor disagree	5 (4.6)
Disagree	0 (0.0)
Strongly disagree	0 (0.0)
Should pay for POCUS training …	
RAMQ	51 (47.2)
Physician	34 (31.5)
Hospital	15 (13.9)
Community	0 (0.0)
Other	8 (7.4)
Should pay for the POCUS device …	
RAMQ	11 (10.2)
Physician	0 (0.0)
Hospital	94 (87.0)
Community	0 (0.0)
Other	3 (2.8)

*AAA* abdominal aortic aneurysm, *CAEP* Canadian Association of Emergency Physicians, *CEUS* Canadian Emergency Ultrasound Society, *EP* emergency physician, *RAMQ Régie de l’assurance maladie du Québec*

## Discussion

The results of this study suggest that some of the CAEP’s recommendations for POCUS use have been implemented in rural EDs in Quebec. They include availability of the device, scope of practice and training. Almost all rural physicians in Quebec EDs have access to a bedside ultrasound device, and a majority of physicians reported using it. The reported rates of POCUS access and use are significantly higher than the rates reported in the Flynn et al*.* study [14], in which only 60.6 % of rural physicians had access to a POCUS device in the ED, and only 44.4 % were trained to use it. One possible explanation for the inconsistent findings is that the Flynn et al*.* study [14] was conducted in 2010. A recent increase in POCUS training initiatives in residency programs may have contributed to increased use by new graduates over the past three years. This hypothesis is supported by the finding that rural physicians in Quebec were younger (median age 37 vs. 49) and were more likely to be recent graduates (average of 7 versus 15 years of clinical experience) than were Ontario physicians. Flynn et al*.* also reported that newer graduates reported greater POCUS use than did more experienced physicians [14].

It is likely that the number of emergency physicians using POCUS in Canada will continue to increase. As per Kim et al*.* [12], nearly all Canadian Emergency Medicine Programs now include POCUS training in their curriculum. All Royal College emergency medicine programs and 88 % of Certificate in Special Competence in Emergency Medicine (CFPC-EM) programs include training in POCUS [9].

Nevertheless, we are surprised by the considerable access to and use of POCUS in Quebec’s rural EDs at this time. Future studies are warranted to examine POCUS use in rural EDs in other Canadian provinces. To date, only a few studies have examined POCUS use in urban settings [23–25]. Comparisons between urban and rural use would be of interest, particularly in the context of reported increased use of CT scanners in academic centers [26].

### Strengths and limitations

Although the 52 % participation rate in the current constitutes a limitation, such rates are commonly reported rate in online surveys of physicians [22, 27]. Furthermore, the participation rate in the present study is significantly higher than the 28 % response rate reported in the only other published report on POCUS use among Canadian rural physicians [14]. The possibility of response bias from enthusiastic POCUS users cannot be ruled out, and must be taken into consideration in interpretation of the results. The study’s design did not allow us to gather data allowing comparisons between responders and non-responders, nor did we inquire about reasons for non-participation. Some of our e-mail invitations may have been lost in junk e-mail folders or sent to an incorrect address. Physician vacation or leaves of absence may have further decreased the participation rate. Our limited response rate despite multiple automated e-mail reminders and personal contacts with ED chairs may reflect the inherent challenges of conducting research in remote and non-academically affiliated centres. Although the survey questions were straightforward and the translated version was pre-tested on francophone residents, we cannot exclude the possibility that some elements were lost in translation. Finally, this study measured self-reported POCUS use; quality of POCUS use and impact on patient care were not determined.

The primary strength of this study is that the sample was composed of physicians working in hospitals in confirmed rural facilities participating in an ongoing study of every rural ED in Quebec. Although Quebec is Canada’s second largest province, future studies are required to determine actual POCUS use in rural settings across Canada and in other countries.

## Conclusion

POCUS use in rural EDs in the province of Quebec seems to be widespread and perhaps more common than in the province of Ontario. Improving access to training programs for rural emergency physicians may further increase POCUS use. Finally, further studies should explore CAEP recommendations that were not included in this study, such as leadership, self-governance, documentation, quality improvement, continuing medical education and research.

## Abbreviations

CAEP: Canadian Association of Emergency Physicians
CCFP-EM: Canadian College of Family Physicians – Emergency Medicine
CEUS: Canadian Emergency Ultrasound Society
CSSS: Centre de Santé et des Services Sociaux
CT: computed tomography
ED: Emergency Departments
FRCP: Royal College of Physicians and Surgeons of Canada
POCUS: point-of-care ultrasound
